# Comparison of Navigated and Frame-Based Stereotactic Biopsy—A Single-Center Cohort Study

**DOI:** 10.3390/medicina60060949

**Published:** 2024-06-07

**Authors:** Maciej Laskowski, Piotr Paździora, Bartłomiej Błaszczyk, Małgorzata Gola, Marcin Ciekalski, Agnieszka Koperczak, Setlak Marcin, Mikołaj Zimny, Anna Zioła-Paździora, Maciej Wojtacha, Adam Rudnik

**Affiliations:** 1 Student Scientific Society, Department of Neurosurgery, Faculty of Medical Sciences in Katowice, Medical University of Silesia, 40-055 Katowice, Poland; s63488@365.sum.edu.pl (M.G.); s76402@365.sum.edu.pl (M.C.); s77599@365.sum.edu.pl (A.K.); 2 Department of Neurosurgery, University Clinical Center, Faculty of Medical Sciences in Katowice, Medical University of Silesia, 40-055 Katowice, Poland; ppazdziora@sum.edu.pl (P.P.); bblaszczyk@sum.edu.pl (B.B.); marcin.setlak@sum.edu.pl (S.M.); d201129@365.sum.edu.pl (M.Z.); mwojtacha@uck.katowice.pl (M.W.); adam.rudnik@sum.edu.pl (A.R.); 3 Department of Pediatric Surgery, Upersiliesian Children’s Health Center in Katowice, 40-752 Katowice, Poland; aziola@gczd.katowice.pl

**Keywords:** brain tumor biopsy, sterotaxy, frameless

## Abstract

*Background and Objectives*: As brain lesions present complex diagnostic challenges, accurate tissue sampling via biopsy is critical for effective treatment planning. Traditional frame-based stereotactic biopsy has been complemented by navigated biopsy techniques, leveraging advancements in imaging and navigation technology. This study aims to compare the navigated and frame-based stereotactic biopsy methods in a clinical setting, evaluating their efficacy, safety, and diagnostic outcomes to determine the optimal approach for precise brain lesion targeting. *Materials and Methods*: retrospective analysis was conducted on patients who underwent brain biopsies between January 2017 and August 2023 at an academic medical center. Data on patient demographics, clinical characteristics, biopsy technique (navigated vs. frame-based), and outcomes including accuracy, complications, and hospital stay duration were analyzed. *Results*: The cohort comprised 112 patients, with no significant age or gender differences between groups. Symptoms leading to biopsy were predominantly diminished muscle strength (42.0%), cognitive issues (28.6%), and aphasia (24.1%). Tumors were most common in the deep hemisphere (24.1%). The median hospital stay was 5 days, with a rehospitalization rate of 27.7%. Complications occurred in 4.47% of patients, showing no significant difference between biopsy methods. However, navigated biopsies resulted in fewer samples (*p* < 0.001) but with comparable diagnostic accuracy as frame-based biopsies. *Conclusions*: Navigated and frame-based stereotactic biopsies are both effective and safe, with comparable accuracy and complication rates. The choice of technique should consider lesion specifics, surgeon preference, and technological availability. The findings highlight the importance of advanced neurosurgical techniques in enhancing patient care and outcomes.

## 1. Introduction

Brain biopsy (BB) has become one of the most important surgical procedures in the management of patients with suspected brain neoplastic lesions. It is also one of the most frequently performed procedures in neurosurgical centers [1]. BBs are used to obtain tissue for histological and genetic examination. Confirmation of the diagnosis guides management of patients with brain lesions. The aim of a brain biopsy is to obtain a small sample of brain tissue for examination, which can help in diagnosing the nature of the lesion and guiding appropriate treatment [2]. Additionally, it can be requested to confirm diagnosis of neurological diseases such as dementia-related disease, infections, or inflammatory conditions [3]. Various surgical methods have been described. Typically, neuronavigation or stereotaxy is used. To precisely and safely access certain regions of the brain, the procedure makes use of imaging technologies like computed tomography (CT) or magnetic resonance imaging (MRI) [4].

Along with the development of brain imaging, the possibilities of biopsy trajectory planning improved, which contributed to an increase in the percentage of successful biopsies and reduction of the risk of complications [5]. However, it should be remembered that the development of imaging techniques may lead to the replacement of the biopsy procedure by, e.g., spectroscopy which, thanks to rapid development, is increasingly more often able to make a diagnosis based on the MR examination alone. Moreover, some studies suggest that Raman spectroscopy could be an effective and accurate tool for differentiating glioma and meningioma from normal brain tissue [6].

BB can be performed by craniotomy, burr hole, twist drill, or endoscopic technics. Image guidance is usually required. Typically, neuronavigation or stereotaxy is used. Since Horsley and Clarke established stereotactic calculation based on a coordinate system and developed a frame fixed to the skull that could precisely locate any intracranial point in three dimensions (3D), that has been the goal of surgery [7].

Stereotactic biopsy (SB) is a minimally invasive technique that is widely used for obtaining an intracranial lesion tissue sample. This method evolved from the Horsley and Clarke stereotactic concept presented in 1908, when they used a stereotactic apparatus to study the deep cerebellar nuclei in experimental animals. SB can be performed with little risk, as tissue can be extracted from foci with a precision of 1 mm and 3 mm in diameter; moreover, pathologic tissue can be histologically compared to surrounding tissue. The biopsy is performed using a stereotactic frame that is attached to the patient’s head. The safest approach to the pathological lesion is established using planning and magnetic resonance imaging, and then computer-defined coordinates are set on the frame. A planned trajectory is used to place the biopsy needle into the skull through a small trepan puncture hole [8].

BB assisted by an intraoperative neuronavigation system includes a camera and computer station with a screen. Neuronavigation allows to track the position of the needle tip in relation to anatomical structures on the screen. To track the position of any surgical tool’s tip during the procedure, this method uses a three-dimensional computer model of the patient’s brain. This allows for an approach that reduces the risk of complications. This method integrates functional data from imaging techniques like fMRI and magnetoencephalography (MEG) to meticulously plan and execute surgical interventions, avoiding sensitive brain regions. Studies have detailed how neuronavigation can be paired with systems such as VarioGuide to enhance the safety and accuracy of stereotactic brain biopsies. This synergy between neuronavigation and intraoperative imaging like MRI has been shown to improve the diagnostic yield of biopsies significantly, ensuring that the biopsy needle is precisely placed, which is confirmed by intraoperative imaging, without increasing the risk of postoperative complications [4,9].

BB procedures are essential for determining the nature of brain disorders, but they come with inherent risks. Complications following these procedures can range from minor to severe, with some leading to long-term consequences or even mortality. Studies have quantified these risks, noting that complications can follow as many as 25.4% of biopsies, predominantly as asymptomatic hemorrhages. Symptomatic complications are less common, occurring in about 3% of cases, and fatalities are rare, at approximately 0.8% [10]. The procedure’s minimally invasive nature belies the potential for significant morbidity and mortality, with rates varying between 0.6% and 6.8% across different studies [2]. Stereotactic brain biopsies, a subset of these procedures, have their own set of risks, though serious adverse effects like death or permanent neurological impairment are deemed rare [11]. The most frequently reported complication is intracranial hemorrhage, with perioperative rates reported as widely as 1.3% to 59.8%. This variance points to differences in surgical technique, patient populations, and possibly the classification of hemorrhages themselves, which can be symptomatic or asymptomatic [12]. The study by Riche et al. found that individuals with glioblastoma or lymphoma, along with older patients, faced a greater likelihood of complications following a brain biopsy. Furthermore, lesions located in the brain stem were more often linked to post-biopsy fatalities, whereas superficial brain lesions tended to have a lower incidence of complications after the procedure [10].

The study aims to evaluate and compare the effectiveness, safety, and diagnostic accuracy of navigated versus frame-based stereotactic biopsy techniques. This comparative analysis seeks to identify the optimal method for precise targeting of brain lesions. A retrospective cohort study was conducted, encompassing patients who underwent brain biopsies between January 2017 and August 2023 at a tertiary academic medical center. The investigation focuses on patient demographics, clinical presentations, biopsy modalities, and outcomes, including successful tissue sampling, complication rates, and length of hospital stay. The primary objective is to ascertain which biopsy technique offers superior diagnostic yield, minimizes complication rate, and enhances overall patient management in the context of neurosurgical interventions for brain lesions. In this study, we also aimed to identify the factors that may influence the occurrence of peri-biopsy complications. By analyzing patient data, clinical characteristics, and procedural details, we sought to determine which variables are associated with an increased risk of complications during and after the biopsy. Understanding these factors is crucial for improving patient safety and outcomes, as it allows for better risk stratification and more informed decision-making in the selection and execution of biopsy techniques.

## 2. Materials and Methods

### 2.1. Patient Selection and Inclusion Criteria

This retrospective, single-center cohort study included all patients who underwent needle brain biopsy between January 2017 and August 2023 within an academic medical center. Clinical information about the patients was registered and documented, including age, sex, primary disease, symptoms, risk factors, number of samples taken, length of hospital stay, information whether the biopsy was successful, location of biopsy, type of biopsy, surgical procedure, surgery-related complications, and early in-hospital complications. We excluded all patients without complete data or follow-up information. The final study group consisted of 112 patients, where 48.2% were female and 51.8% male patients, with the average age of 58. Symptoms were divided into groups including seizures, headaches, behavioral disturbances, altered levels of consciousness, memory impairments or cognitive dysfunctions, speech disturbances, aphasia, dizziness, muscle weakness, partial paralysis, sensory, visual disturbances, and ataxia. We acquired data about risk factors that included hypertension, type II diabetes, atherosclerosis, hyperlipidemia, venous thrombosis, cancer other than CNS, and hypo- and hyperthyroidism.

### 2.2. Indications for Surgery and Surgical Procedure

The indications for surgery were all unknown brain lesions for a histopathological diagnosis—which have not been qualified for tumor resection in first place, or patients with multiple systemic tumor diseases and new brain lesions. Patients underwent one of two surgical aspiration biopsy procedures using either the stereotactic needle brain biopsy (Lexell-based stereotactic frame and Innomed ZD) or neuronavigated needle brain biopsy based on surface matching (Medtronic Vertek Passive Biopsy, Stealth Station). Magnetic resonance imaging (MRI) with navigation sequencing was performed on all patients prior to surgery. A biopsy was considered successful if it allowed adequate tissue sampling for performing histopathological, and in certain cases genetic, testing. The diagnostic yield of effective biopsy was enough to conduct adequate clinical management for a patient. A successful biopsy correctly identifies the nature of the brain abnormality, whether it is a tumor, infection, inflammation, or other conditions.

### 2.3. Postoperative Management

Following the procedure, every patient underwent a postoperative CT to check for any immediate complications. After the histopathological results were available, a multidisciplinary Tumor Board comprising neurosurgeons, radiation therapists, neuro-oncologists, and neuroradiologists reviewed each case to provide recommendations on subsequent surgical interventions. The consecutive treatment plan was based on histopathological results, follow-up MRI, and clinical status assessment. In the case of a successful needle biopsy, the follow-up treatment consisted of surgical resection, radiosurgery, whole-brain radiation, chemotherapy, or palliative treatment. In the case of unsuccessful procedures, the next clinical steps were based on the clinical state of a patient and his or her will to continue treatment. In the event of a significant increase in mass effect or sampling of necrotic tissue, the treatment plan was shifted from needle biopsy to microsurgical resection or cytoreduction. In the case of severe clinical deterioration, palliative treatment was recommended.

### 2.4. Ethical Standards

The methodology and procedures were performed in accordance with the ethical standards established by the 1964 Declaration of Helsinki and its subsequent amendments.

### 2.5. Statistics

The analysis of the data collected was performed using Statistica 13.0 software (StatSoft, Krakow, Poland). Categorical variables were represented as numbers or percentages and compared using Chi-square tests. Continuous variables, expressed as means ± standard deviation or as mean with lower and upper quartiles, were analyzed using either the Student *t*-test or the Mann–Whitney U test, depending on the normality of the distribution. The Shapiro–Wilk test was employed to assess the normality of result distribution. The correlation among the examined qualities was tested using Spearman’s rank correlation coefficient. A significance level of less than 0.05 was applied to all tests.

## 3. Results

Following data acquisition, 112 patients were incorporated into our study, with 58 (51.8%) of patients being male. The mean age at hospital admission was 57.96 ± 14.76 years. The age and ratio of female to male patients did not show a significant difference in either group (Table 1). Diminished muscle strength (42.0%), cognitive and memory problems (28.6%), and aphasia (24.1%) were the primary symptoms reported by patients. The prevailing comorbidities included arterial hypertension (47.3%), diabetes mellitus (17%), and hyperlipidemia (12.5%). Table 2 presents a complete list of patient symptoms and health issues.

Tumors predominantly affected the white matter of telencephalon (24.1%), with subsequent involvement in the frontal (21.4%), temporal (16.1%), and parietal (11.6%) lobes. The brain stem (2.7%) and cerebellum (2.7%) were the least affected regions (Table 3). Disparities in localization between groups were evident in the frontal lobe (navigated 19 (25.33%), stereotactic 5 (13.51%)), temporal lobe (navigated 10 (13.33%), stereotactic 8 (21.62%)), and occipital lobe (navigated 6 (8%), stereotactic 0). Comparisons of biopsy type with localization are particularly described in Table 4. The procedure choice was based on surgeons’ preference.

The most significant disparity in biopsy outcomes was noted in tumors situated in the temporal area (*p* = 0.088) (Table 4). Spearman correlation analysis revealed a weak negative correlation between navigated occipital biopsy with successfulness of procedure (*r* = −0.24) and negative correlation between temporal stereotactic biopsy with success rate of the procedure (*r* = −0.42).

The duration of the first hospitalization displayed diversity, with a median of 5 days (interquartile range, Q_1_ = 4; Q_3_ = 7). The most prolonged hospital stay, lasting 25 days, was linked to complications associated with bleeding. Most patients (72.3%) were not rehospitalized. The most common reason for rehospitalization was tumor resection (11.6%), while the least common was valve implantation in treatment of acute hydrocephalus (1.8%) and wound care/fistula (1.8%) (Table 5).

Overall, five patients (4.47%) experienced early postoperative complications, and there was no significant difference between the frame-based and navigated groups. Specifically, bleeding complications were observed in four patients (two each in the navigated and stereotactic groups), and one patient developed a fistula (in the stereotactic group). Chi-square, unpaired *t*-tests, and the Mann–Whitney U test did not reveal any significant differences between analyzed groups, except for a statistically significantly lower number of biopsy samples collected from the navigated biopsy procedure (*p* < 0.001). Detailed *p*-values for each analyzed characteristic can be found in Table 1. In the case of biopsy efficiency in tissue sampling, 30 (81.1%) stereotactic biopsies were successful, 7 (18.9%) were not, 61 (81.3%) navigated biopsies provided adequate lesion sampling for histopathologic report, and 14 (18.7%) did not yield diagnostic value. Twelve (10.7%) patients received secondary biopsy due to inadequate histopathological results. In four (3.5%) cases of ineffective needle biopsy, the Tumor Board decided to transfer patients into palliative care due to clinical deterioration. Follow-up MRI revealed rapid progression of mass effect in three (2.7%) of the patients. For those cases, the treatment plan was modified into microsurgical resection. Two (1.8%) cases were lost to follow-up due to the patients’ decision to withdraw treatment.

Patients were categorized into four groups based on their histopathological diagnoses (Table 6). Group 1 comprised individuals diagnosed with diffuse large B-cell lymphoma. Group 2 consisted of patients with gliomas graded as WHO I and II. Group 3 included patients diagnosed with gliomas graded as WHO III and IV. Group 4 comprised patients with schwannomas, metastases, SM, inflammation, and those lacking malignant features in biopsy. Spearman correlation analysis indicated weak positive correlations between Group 1 and Group 3 with successful brain biopsy (*r* = 0.22 and *r* = 0.21, respectively), while Group 4 exhibited a negative correlation (*r* = −0.38). Additionally, Group 3 demonstrated a weak positive correlation with longer hospitalization (*r* = 0.20).

## 4. Discussion

We compared the effectiveness of both types of biopsies by estimating the number of specimens taken in both biopsies and assessing the diagnostic accuracy of the specimens, or more precisely, whether there was a need for a repeat biopsy.

A correlation test of biopsy type with its accuracy showed no significant differences, where 30 stereotactic biopsies were successful, 7 were missed, 61 navigated biopsies were successful, and 14 were missed. The “*n*” denotes the number of cases or instances in each category. Drawing on 15 incorporated studies, Dhawan et al. determined that existing research indicates that navigated stereotactic brain biopsies are equally as effective as frame-based methods when considering diagnostic precision and the occurrence of negative clinical events [13]. Another meta-analysis performed by Kesserwan at al. that encompasses five extra studies, employing a broader search approach, reinforces the conclusions of earlier research findings [14].

This study is mostly focused on supratentorial lesions due to the low number of infratentorial cases. Performing needle biopsy in the posterior fossa is challenging but can be performed with good results [15]. Needle biopsies in the posterior fossa are less frequently used due to the facts of lower incidence of posterior fossa tumors in the adult population and the common situation of IV ventricle compression, which limits the appliance of radiosurgery. In case of planned microsurgical tumor resection, performance of a needle biopsy before definitive treatment is questionable.

We also estimated that the median number of specimens in stereotactic biopsy was six and in the navigated five. In navigated brain biopsies, a study by Vychopen et al. focusing on patient safety compared navigated and stereotactic and found that, on average, three tissue samples were taken per biopsy, regardless of the method used. This consistency suggests a standard practice in the amount of tissue required for effective diagnosis [16]. In contrast, stereotactic brain biopsies often involve collecting a larger number of specimens. The average number of specimens provided in these procedures is around 15, with 5 being typically used for molecular genetics diagnostics. Another source cited an average of 15 to 16 specimens. This higher number might be attributed to the need for more comprehensive sampling in certain cases, or the nature of the stereotactic technique, which allows for precise targeting of different regions within the lesion [17].

It has been conventionally hypothesized that the quantity of specimens acquired during a brain biopsy may influence the risk of morbidity and mortality. Nevertheless, our investigation found no evidence to substantiate this correlation. It is critical to acknowledge the limited scale of the patient group when interpreting our findings. A study addressing the clinical outcomes related to the number of biopsy samples taken during brain biopsies found that morbidity rates were 4.3% for less than three samples, 16.3% for three–six samples, and 17% for more than six samples. These rates were statistically significant. Despite the variation in morbidity, the diagnostic yields were comparable across the different groups, regardless of the number of samples taken [18].

The complication rate might also be dependent on the tumor type. In this paper, no differences have been found between each tumor subtype. A study conducted by N. Sugii concluded that an increased risk of bleeding is a case of high-grade glioma in compression with low grade [19].

It is noteworthy that in our study group, out of 112 patients, there were only 5 complications, suggesting a relatively low complication rate. This incidence of complications, predominantly hemorrhages, aligns with known risks associated with brain biopsy procedures [11]. The occurrence of a single fistula and the absence of any infections can be seen as indicative of stringent aseptic techniques and effective perioperative care. However, in our study, a single fistula was associated with the occlusion of the burr hole using bone wax. Bone wax is usually applied along the edges of the bone to inhibit bleeding after surgery. In the fistula case, bone wax was used to fully close the burr hole. In our opinion, excessive use of bone wax is associated with increased risk of fistula formation. The results indicate that there were no significant differences in complications based on the type of biopsy when assessed with a Chi-square test. Specifically, for stereotactic biopsies, there were 3 instances of complications and 34 without complications. For navigated biopsies, there were 2 cases with complications and 73 without.

The study’s findings contribute to the ongoing assessment of brain biopsy safety, emphasizing that both navigated and stereotactic techniques, when performed with skill and precision, result in a low rate of adverse events. These outcomes should be considered alongside other studies to better understand the risk profile of these neurosurgical interventions. The rarity of severe complications reinforces the role of meticulous surgical planning and execution, as well as the importance of postoperative monitoring to identify and manage complications promptly. Future discussions could benefit from a comparative analysis of long-term outcomes and the functional impacts of these complications, providing a more comprehensive understanding of the patient recovery trajectory following brain biopsies.

Tumor location may affect the success rate of biopsy procedure. Different authors present various insights into this matter [16,20]. The performed study reveals the lowest success rate for occipital lobe biopsies. Procedures in this localization require advanced patients positioning on the side, prone, or semi-sited. Surface registration is exposed to inaccuracy because of it.

Success rates for frame-based and frameless are similar if we compare all procedures. A closer look at the success rate in comparison to the specific tumor location reveals interesting insight. Positive surgical results might be more affected by choosing the appropriate technique for different locations. Even though the results did not reach statistical significance, lower rates of surgical failure have been observed for frameless biopsy in the temporal region and the frame-based technique in regions of white matter of telencephalon (deeper biopsies). This finding might be attributed to the fact of placement of a stereotactic frame which is usually placed at the level of the temporal lobe. This type of placement might affect the choice of trajectory and be a reason for selecting a suboptimal route for stereotactic biopsy in temporal region lesions. On the other hand, biopsy of deeper located tumors showed a higher success rate for stereotactic procedures. This result may be caused by higher target error. The mean target error for the frame-based procedure is 0.9 ± 0.3 mm [21]. The mean target error for frameless procedures is 2.7 ± 0.56 mm [22]. Otherwise, the shift between a planned and actual target might not be important from a clinical point of view in matters of successful tissue sampling due to the fact of much bigger size of typically treated lesions. It might be more important to plan a biopsy site based on PET molecular imaging, for example [23]. Frameless procedures are suspected of lower accuracy due to the surface registration process with risk of skin compression and movement during this process. Similar results for successful procedures seem to refute this hypothesis.

Even though the results and safety profile are similar between frame-based and frameless procedures, there are important differences in the application technique. Patients planned for brain lesion biopsy require preoperative MRI/CT for surgical planning [24]. Stereotactic procedures usually require a second imaging study, usually CT after placement of the frame. Due to this fact, performing a frame-based biopsy consumes more time from the surgical team and requires additional radiation exposure of the patient. A longer procedure time and placement of frame under local anesthesia seems to also affect patient comfort due to the pain and discomfort related to placement of local anesthesia and increased weight of the head with the frame applied. In our study, all of the stereotactic biopsies were performed under local anesthesia, in contrast to neuronavigated procedures, which were almost only performed under general anesthesia. Intubation of patient after placement of a stereotactic frame is challenging. Due to this fact, performing the frame-based biopsy under general anesthesia requires prolonged general anesthesia and anesthesiologic work. In contrast, a frameless biopsy under general anesthesia requires a lot less work time from the anesthesia team, even though the surgical time is similar in both procedures. In some cases of frameless biopsy, the choice of local anesthesia was dictated by poor cardiopulmonary status of the patient.

Another important aspect of comparison of those methods is the cost of treatment. Both procedures require similar hospital stay operating theater time. The major difference of procedure cost might be related to single-use equipment needed for surgery. The frameless biopsy technique consumes markers used for visualization of anatomy during procedure, and a biopsy needle is a single-use piece of equipment. On the other hand, stereotactic intervention is performed with instruments which can be sterilized. As mentioned above, even though the surgical time is similar, preparation for a frame-based procedure requires much more time from the surgical team. In order to perform adequate analysis of procedure costs, additional research should be conducted.

## 5. Conclusions

Our study comparing navigated and frame-based stereotactic brain biopsies with tissue sampling below eight per case reveals equivalent efficacy and safety between the two methods. Both are effective and safe for diagnosing brain lesions, demonstrating comparable accuracy and low complication rates. This suggests that the choice of technique can be based on case specifics, surgeon preference, and available technology, rather than any inherent superiority. The operational difference, notably the lower number of samples collected via navigated biopsy, did not impact diagnostic outcomes. These findings highlight the importance of precision and quality of biopsy samples over quantity. Future research should focus on optimizing biopsy techniques and integrating emerging technologies to enhance accuracy and safety further. This study supports a tailored approach to selecting biopsy methods, emphasizing the need for clinical decision-making grounded in patient-specific and lesion-specific considerations.

## Figures and Tables

**Table 1:** Comparison of patients depending on biopsy type.

Characteristic	All Biopsies	Navigated	Stereotactic	*p*-Value	No Data (*n*)
Total (%)	112 (100)	75 (66.96)	37 (33.04)	-	0
Mean age (SD)	57.96 (14.76)	58.77 (14.67)	56.50 (15.02)	0.462	11
Gender, F:M	54:58	39:36	15:22	0.254	0
Complications (%)	5 (4.47)	2 (2.67)	3 (8.11)	0.201	0
Successful brain biopsy (%)	91 (81.25)	61 (81.33)	30 (81.08)	0.974	0
Median number of biopsy samples (Q_1_–Q_3_)	5 (4–6)	4 (3–6)	6 (5–7)	<0.001 *	0
Number of days in hospital during first biopsy (Q_1_–Q_3_)	5 (4–7)	5 (4–6)	5 (4–7)	0.972	2
Number of hospitalizations (Q_1_–Q_3_)	1 (1–2)	1 (1–3)	1 (1–2)	0.711	2

SD—standard deviation, F:M—female-to-male ratio, * statistically significant.

**Table 2:** Patient symptoms and comorbidities.

		*n* (%)
Presenting symptoms	Diminished muscle strength/Paresis/Plegia	47 (42.0)
	Cognition and memory problems	32 (28.6)
	Aphasia	27 (24.1)
	Headache	25 (22.3)
	Behavioral disorders	21 (18.8)
	Dizziness	19 (17.0)
	Epilepsy	15 (13.4)
	Visual impairment	14 (12.5)
	Ataxia	14 (12.5)
	Confusion	14 (12.5)
	Sensory deficit	6 (5.4)
Comorbidities	Arterial hypertension	53 (47.3)
	Diabetes mellitus	19 (17.0)
	Hyperlipidemia	14 (12.5)
	Hypothyreosis	14 (12.5)
	Other neoplasia	12 (10.7)
	Atherosclerosis	8 (7.1)
	Venous thrombosis	4 (3.6)

**Table 3:** Tumor locations of patients undergoing brain biopsy.

Location	*n* (%)
Frontal	24 (21.4)
Temporal	18 (16.1)
Parietal	13 (11.6)
Occipital	6 (5.4)
Deep part of hemisphere	27 (24.1)
Corpus callosum	8 (7.1)
Insula/thalamus	5 (4.5)
Ventricle	5 (4.5)
Brain stem	3 (2.7)
Cerebellum	3 (2.7)

**Table 4:** The success of biopsies, categorized by type and location.

Location	Navigated		Stereotactic		*p*-Value
	Successful	Unsuccessful	Successful	Unsuccessful	
Frontal	18	1	4	1	0.38
Temporal	9	1	4	4	0.088
Parietal	7	1	4	1	0.641
Occipital	3	3	0	0	-
White matter of telencephalon	13	5	8	1	0.323
Corpus callosum	4	2	2	0	0.536
Insula/thalamus	2	0	3	0	-
Ventricle wall	3	0	2	0	-
Brain stem	1	1	1	0	0.667
Cerebellum	1	0	2	0	-
Total	61	14	30	7	0.974

**Table 5:** Factors leading to the patient’s rehospitalization.

Factors	*n* (%)
Absence of rehospitalization	81 (72.3)
Resection	13 (11.6)
Secondary biopsy	12 (10.7)
Acute hydrocephalus/valve implantation	2 (1.8)
Wound care/fistula	2 (1.8)

**Table 6:** Comparison of patients depending on histopathological categorization.

Characteristic	Group 1	Group 2	Group 3	Group 4
Total (%)	19 (16.96)	17 (15.18)	44 (39.29)	30 (26.79)
Mean age (SD)	62.87 (11.89)	48.76 (16.88)	60.68 (12.22)	57.79 (14.83)
Gender, F:M	11:8	9:8	18:26	16:14
Biopsy type, N:S	16:3	10:7	28:16	20:10
Complications (%) ^α^	2 (10.53)	0 (0)	1 (2.27)	1 (3.33)
Successful brain biopsy (%)	19 (100)	13 (76.47)	40 (90.91)	17 (56.67)
Number of days in hospital during first biopsy (Q_1_–Q_3_)	4 (3–6)	5 (4–5)	5 (4–7)	6 (4–7)

^α^—Two patients (one with complications) lacked a definitive histopathological diagnosis, leading to their exclusion from the analysis, SD—standard deviation, F:M—female-to-male ratio, N:S—navigated-to-stereotactic ratio.

## Data Availability

Data are contained within the article.
